# Unmanned aerial vehicles and pre-hospital emergency medicine

**DOI:** 10.1186/s13049-024-01180-7

**Published:** 2024-01-29

**Authors:** Katy Surman, David Lockey

**Affiliations:** 1 Bartshealth NHS Trust, London, UK; 2 Blizard Institute, Queen Mary University, London, UK; 3 London’s Air Ambulance, Barts Health NHS Trust, London, UK

## Abstract

Unmanned aerial vehicles (UAVs) are used in many industrial and commercial roles and have an increasing number of medical applications. This article reviews the characteristics of UAVs and their current applications in pre-hospital emergency medicine. The key roles are transport of equipment and medications and potentially passengers to or from a scene and the use of cameras to observe or communicate with remote scenes. The potential hazards of UAVs both deliberate or accidental are also discussed.

## Introduction

Innovation in pre-hospital emergency medicine is often introduced via two routes. Firstly, by extending proven in-hospital interventions into pre-hospital practice to address time-critical clinical problems and secondly by using technology and techniques from industries outside medicine to address medical challenges. Unmanned aerial vehicles (UAVs) have been used in many industries for many years for commercial applications and their use has expanded in recent years as technology has improved and costs have decreased. They have been defined as ‘an aircraft piloted by remote control or onboard computers’. The military use of UAVs is reported on a daily basis from ongoing conflicts but applications in medical roles are also progressing at a rapid rate. Although the emergency community may have to prepare for encountering patients harmed by UAV attacks it will also benefit from applications designed to contribute to patient care.

Imaging UAVs have become commonplace in several industries including farming, environmental surveillance, military surveillance and structural surveying. In these roles UAVs are often a much cheaper option than manned aircraft. They can also be used in dangerous, inaccessible or non-permissive environments. Delivery UAVs are being trialled by many commercial organisations and are now able to rapidly deliver small or moderate sized items considerable distances.

Medical applications of UAVs are attracting considerable attention in the medical [1, 2] and general media and have the potential to improve the delivery of care in a number of diverse areas. Some of the applications under investigation, and of interest to the pre-hospital and emergency medical community, include the rapid delivery of defibrillators in out of hospital cardiac arrest (OHCA), the delivery of blood products and medications and the assessment of incidents and major incident scenes. In addition to improving response times to time-critical incidents UAVs can carry a variety of different communication and monitoring equipment to carry out specific roles.

This article will review current roles of UAVs in pre-hospital medicine and emergency responses. In addition, we will address the current challenges and areas for future research and development.

## Methodology

A systemic literature search was performed to review current research and applications of UAVs in pre-hospital medicine. PubMed, WOS, Cochrane, OVID and Embase were searched electronically for relevant articles using relevant terms: (pre-hospital) OR (pre hospital)) OR (emergency)) AND (drone)) OR (UAV)) OR (unmanned aerial). Titles and abstracts were screened for papers of relevance and selected if applicable. Full articles were then reviewed to ensure relevance before being included in review. This search strategy initially yielded 3234 hits. 56 studies were of relevance and 44 were included in the final paper. Of these seven were medical reviews, two medical guidelines, one a technical review, 28 medical original research papers and six technical original research papers. On the basis that UAV development is often reported in the commercial and non-medical literature reference lists from relevant papers were also scanned to include material from outside the medical literature. Sources included academic journals, news articles and electronic resources. A further 32 references were included from reference lists which included 10 medical original research studies, 12 technical original research studies and the remainder from reference documents, media articles and manuals.

### UAV characteristics and capability

The challenges and limitations of UAV applications in emergency medical (and most other roles) are, to a great extent, dependent on the technical specifications of the vehicles and their ability to safely operate in the relevant airspace. Most published studies of UAV utilisation report multirotor UAVs which are used because of their compact size, simple control mechanisms, and ability to hover while performing tasks. Their disadvantages are that they often have a limited load capacity and a range limited by high energy requirements. Fixed-wing UAVs have a very different construction with a body, wings and a motor driving a propeller. This gives them longer range and the potential to carry heavier loads. For this reason, they are often used for transport roles. Disadvantages include the fact that they require a larger landing site and more skills to fly. The type of UAV selected is sometimes determined by the range and flight time limits. Some large UAVs can fly for several hours but are expensive. The uses of smaller UAVs can be limited by flying times which may only be 20–30 min. Flight time is limited by the power supply provided by batteries. Batteries with larger capacity are heavier and this limits flight time. This can be improved with the use of advanced battery design, hybrid systems or the use of internal combustion engines. Even when range and performance are optimised the performance of UAVs can be significantly influenced by harsh environments and adverse weather conditions. Rain, wind and temperature have a major impact on battery endurance and flight times.

A number of technical solutions are being explored to increase the endurance of UAVs although there are currently no published reports of widespread commercial use. These include using solar power, wind power (‘gust soaring’), in-flight charging from overhead power lines and wireless power charging. Other innovations include automated battery swapping from remote sites where a UAV can discharge a spent battery and pick up a new one or automatically dock at a remote site to recharge before a further flight [3].

One of the most challenging aspects of UAV use is deconfliction with other air traffic and compliance with regulatory authorities. This is particularly problematic with emergency use where flights are not planned. Planning for the use, regulation and safe deconfliction of UAV traffic with restrictions on size, air traffic compliance, flight routes and registration of users is rapidly evolving both at national and international level [4]. Regulation is complex and variable although most authorities impose much stricter control on UAVs flown outside the line of sight of the operator and for larger UAVs [5]. Although technical solutions have been proposed [6, 7] they require the coordination and cooperation of authorities and operators with sometimes conflicting aims and objectives.

### Utilising UAVs for transport and delivery

#### Transport and delivery of defibrillators to victims of cardiac arrest

Cardiac arrest outside hospital is common and in Europe the annual incidence is reported to be between 67 and 170 per 100,000 inhabitants. Resuscitation is attempted or continued by EMS personnel in about 50–60% of cases and discharge rates from hospital are very low (an average of 8%). Many initiatives have attempted to improve survival [8] and improving the rate of bystander CPR and the use of automated external defibrillators (AEDs) have been identified as crucial to improving survival. Bystander CPR and early defibrillation doubles the chance of survival and reduces morbidity associated with cardiac arrest [9]. Although there are many public access defibrillator programmes in place to facilitate earlier access to defibrillators, many OHCAs occur in places where defibrillators are not immediately accessible, and novel methods of enabling faster access to defibrillators are required [10].

Studies across Europe have demonstrated the feasibility of UAV transport of defibrillators directly to the patient. In rural areas it has been suggested that UAVs can deliver defibrillators prior to the arrival of land crews in 93% of cases [11] and that delivery of a defibrillator using UAVs can often be quicker than a pedestrian can locate and retrieve a community defibrillator [12]. Although particularly relevant for rural areas, mathematical modelling in both North America and Europe has shown that UAVs also have the potential to reduce time from OHCA to defibrillation in urban areas [13–15]. Modelling in the Toronto region estimated time savings to be around six minutes in urban areas and ten minutes in rural areas using approximately 100 UAVs in the region for defibrillator delivery [13]. In Sweden detailed modelling has been reported to calculate the number of AED– UAVs that would be required to deliver an eight minute response to different proportions of the population [16]. Much of the work reported on defibrillator delivery has been theoretical but in 2021 Schierbeck et al. reported a case of an individual who underwent successful defibrillation with a UAV delivered defibrillator in Sweden just prior to the arrival of a land emergency ambulance team [17].

Prompt delivery of a defibrillator by UAV to a patient in the pre-hospital environment appears to be a realistic and safe option. Delivery may even be possible at night [18]. However, there may be additional work required in educating the public in the effective use of UAV delivered automated defibrillators for OHCA. In a simulation study Rosamond et al. demonstrated that despite a UAV being delivered prior to the arrival of land crews a significant proportion of participants had some difficulties with AED use. [19]. Clear instruction, possibly by telephone dispatchers [20] and training and information campaigns via media and social media may be required to familiarise the public with delivered defibrillators [21–23]. Some initiatives have examined the possibility of delivery to the location of a mobile telephone to ensure an accurate delivery location and also delivering to community first responders to ensure that an effective user is immediately available to operate the defibrillator [24].

Although strong evidence for feasibility is available, many emergency medical service (EMS) systems still need to develop robust and safe dispatch models within aviation regulatory rules as well as ensuring that the AEDs are effectively utilised after successful delivery. The published work on UAV defibrillator delivery has resulted in the European Resuscitation Council and the International Liaison Committee on Resuscitation to comment in their 2021 guidelines [25], on the potential benefits and future work required for practical implementation of this intervention.

#### Transport and delivery of medical supplies

UAVs have the potential to play a role in delivery of time-sensitive medical supplies, including blood products and emergency medications. The exact role and potential benefits of this type of initiative depend very much on the operating environment– the delivery of an emergency medication in an urban medical emergency is very different to the delivery of medical supplies or blood in a remote or difficult access location.

Blood products have become an integral component of early resuscitation in major haemorrhage, both in hospital and in the pre-hospital environment. Both military and civilian studies have demonstrated reduced mortality after early transfusion of blood products in major trauma [26].

Transport of blood products requires careful quality control to ensure that the products are safe for transfusion after delivery. This includes prevention of the adverse effects of temperature variation and prevention of haemolysis when compared to standard land transport [27, 28]. Errors in transport and storage of blood products carries a significant risk for patient safety [29]. Pilot studies have indicated that using UAVs to transport blood products (packed red cells, platelets and frozen plasma) is safe and feasible, with little adverse impact on the products during transport. UAV delivery of blood products has been utilised successfully in both Rwanda and Ghana for many years with a significant reduction in delivery times when compared to standard land transport [30, 31]. Rwanda is particularly suitable for a blood delivery programme because it is a small densely populated country with a poor road infrastructure and a majority of the population living in rural areas. When over 12,000 UAV blood deliveries were evaluated in Rwanda between 2017 and 2019 the mean delivery time was reduced by 79 min to 49.6 min. Significant reductions in blood wastage were also reported [32]. This study also reported internal data by the UAV provider which suggested that of more than 100,000 units of blood delivered over three years less than 1% were damaged during delivery.

The treatment of trauma victims in remote and inaccessible places is a key aspect of military medicine. Delivery of blood to medics treating military casualties has been considered for a number of years and a recent review has summarized current capability [33]. Various military simulations, some involving delivery of real blood products, have indicated that UAV delivery is not only practical but also maintains the quality of blood products delivered [34, 35].

The administration of medication in time critical conditions can be life saving. This may be particularly effective where medications can be administered by non-medical personnel. Examples include epinephrine for anaphylaxis, anticonvulsants for prolonged seizures and naloxone for opiate overdose. UAVs offer the potential to reduce time to access lifesaving medications and computer modelling has shown the potential for a significant reduction in time to delivery of these medications compared to standard land crew delivery [33–35].

Although not strictly emergency medicine related but demonstrating logistical feasibility, UAVs have also been shown to be effective in transporting chemotherapeutic agents which typically have a short shelf-life, to rural and remote areas, providing access to these medications in areas where it was previously logistically challenging [36]. In common with blood, there are quality control factors to consider when transporting and administering medications. These may include stable storage temperature and vibration during the transportation process and safe use of potentially harmful or controlled drugs. In terms of safe administration, a feasibility study performed in the US has demonstrated that bystanders were able to successfully carry out instructions given by the emergency call handlers to retrieve UAV-delivered intranasal naloxone and administer this safely to the patients whilst awaiting arrival of land emergency medical teams [37]. Although further studies are required to confirm the safety of administration of medication in this manner, this does support the real possibility of layperson administration of life-saving medication delivered by UAVs. Other useful medications delivered in specific time critical conditions (e.g. bronchodilators for asthma or snake antivenom in inaccessible sites) may also justify future investigation.

The challenges of the introduction of medical UAV delivery systems need to take into account many factors before implementation and are very specific to regional demand and conditions. An example of the scope of factors that need to be considered is demonstrated in a concepts paper published in 2019 [38] which examined potential medical UAV uses in Madagascar, Malawi and Senegal. The challenges were extensive and included the provision of robust and simple UAVs to be used in difficult climatic conditions with a limited communications infrastructure and challenging aviation regulatory legislation. The paper provides recommendations on key areas which need to be addressed in setting up a UAV service including regulatory, feasibility studies, acceptability by the target population and how to monitor and evaluate the implementation. These recommendations are targeted at service providers, governments, UAV providers and potential funders.

#### UAV Transport of patients

Evacuation of combat casualties to medical care in a timely manner has long been a key objective of military medicine and continues to influence modern military policy [39]. The use of UAVs to evacuate patients from a non-permissive environment has been extensively discussed in recent years [40]. Challenges include the provision of UAVs with the capacity to carry a heavy load and the possibility of a casualty having to fly without medical supervision. Larger UAVs with the capacity to carry heavy loads, are currently available, particularly for military applications although they are expensive. Prototype UAVs with the capacity to carry two adults in a military environment have been reported for several years [41]. A recent study has also confirmed that many military casualties do not require critical interventions during evacuation and might therefore be suitable for unsupervised UAV evacuation [42]. Although intervention may not be possible during evacuation remote monitoring devices are available and receiving centres could have real-time physiological observations and be able to observe a patient’s condition prior to arrival. There have been recent reports of remotely operated military helicopters completing simulated casualty evacuations without incident [43] and it seems likely that pilotless aircraft will soon be part of military medical practice. Although casualty evacuation by UAV has been mostly theoretical and developmental there are now reports of real casualties being evacuated by UAV in the Ukraine conflict [44]. Military requirements have often driven progress in civilian trauma medicine and these developments may be significant in understanding and developing UAV potential in civilian evacuation pre-hospital practice.

### UAVs and roles in surveillance

#### Telemedicine

As UAVs can be controlled and monitored remotely, they have the potential to assist in providing emergency telemedicine for patients with limited or delayed access to emergency medical care. This may be particularly helpful for patients in areas that are in remote or non-permissible environments. Two-way video communication can assist diagnosis, evaluation and guide intervention, such as advising bystanders on how to perform cardiopulmonary resuscitation. Sensors incorporated into UAVs can monitor basic physiology [45], such as respiratory rate, temperature, blood pressure, heart rate and peripheral oxygen saturations which may provide useful extra information to assist remote, assessment and initial management. There is even potential for UAV monitoring of cardiorespiratory signs without the need for landing or physical contact with the patient [45]. Video cameras installed on UAVs may be able to detect facial movements and respiratory movements to assess for signs of life [46]. This information can be helpful in triage or rescue resource allocation. Video scene assessment can help identify the number and location of casualties and identify scene hazards prior to rescue.

#### Search and rescue

The use of UAVs in search and rescue services is becoming increasingly common to locate missing persons in remote and rural areas. There are a number of different technologies that can assist UAVs in performing this role. Live video cameras, thermal imaging cameras and multispectral analysers can be all be carried to contribute to earlier detection of missing individuals. UAVs are currently in use in many emergency services to assist in the location of missing hikers or skiers in mountainous or avalanche areas. Some mountain rescue teams have suggested that this technology can reduce time to location of avalanche victims by up to 50% [47, 48].

Drowning is another time critical rescue challenge. Favourable neurological outcomes decrease rapidly after even a short submersion time. UAVs can assist in providing earlier identification of swimmers in trouble when compared to traditional search techniques which increases the liklihood of earlier rescue and survival [49]. In addition, UAVs have been demonstrated to effectively deliver flotation devices significantly faster to individuals in the water when compared to standard rescue operations, even in poor weather conditions [50, 51].

In less remote conditions advanced first aid systems are being developed for vulnerable elderly patients. This can combine technology that detects falls and then uses UAVs for location with the additional capacity to transport first aid equipment while emergency services are alerted [52].

#### Major incident / Mass casualty responses

Rapid assessment of major incident scenes and determination of the position, number and state of victims is vital to effective incident management. UAVs have been proposed and used to achieve these objectives by many emergency services. They are particularly useful in potentially non-permissive environments before rescuers enter the scene. Smaller UAVs can be rapidly deployed to more contained scenes such as urban or isolated incidents while longer range UAVs may be more suitable for the management of larger natural disasters. The technical feasibility of UAVs in these roles has already been established for searching large areas for hazards such as wildfires in remote areas [53].

A number of simulation studies have been conducted which have suggested that UAVs can be used by relatively inexperienced operators to accurately assess major incidents more effectively than with standard techniques [54]. They can be used to effectively identify scene hazards, assess and triage patients and effectively set up key incident locations [55]. The technology has been reported to be acceptable to incident commanders and is perceived as straightforward to introduce and use [56]. The images can be used for real time management of incidents and to give strategic overview to remote control centres. An example of successful UAV use in a major incident is reported by emergency services after a building collapse in Taiwan in 2018 which killed 115 people [57]. The authors describe how a combination of fixed cameras and UAVs informed scene commanders and allowed the 3D construction of the incident site to plan rescue and casualty extrication.

UAV use can help avoid the exposure of emergency service personnel to hazards and may have a role in chemical, biological, radiological and nuclear (CBRN) events. UAVs have also been developed with the capacity to sample and detect hazardous gases [58] and radiation sources [59] as well as more conventional roles such as delivery of equipment for decontamination and other medical uses to the incident site. Additionally, loudspeakers on UAVs can help incident command teams to communicate with people on the ground. This could allow instructions on decontamination, directing people to areas of safety, or providing instructions for lifesaving interventions, such as compression of bleeding injuries.

UAVs have the potential to be used on a larger scale in disaster management, providing emergency surveillance, equipment delivery, food supplies and identification of missing persons [60]. They have been used in devastating environmental disasters, such as the 2015 earthquake in Kathmandu to identify buildings that may be at risk of collapse, or the direction of flood water, and can help to map safe evacuation routes [61, 62].

#### Triage

It has been proposed that remote triage can be conducted with UAVs. This may easily detect mobile casualties [55] but more advanced techniques may also be able to detect more subtle signs of life in immobile victims [63]. Remote triage algorithms to complement standard triage techniques have been developed and the overall concept has been welcomed by experienced clinicians [64]. New methods of triage are a rapidly evolving area of major incident management and the incorporation of remote techniques utilising UAVs are likely to be an important addition to triage capability.

### UAV use in the COVID 19 epidemic

The recent COVID 19 epidemic stimulated rapid development of technological solutions and research activity in an attempt to overcome unique and severe healthcare challenges. UAV applications were not excluded from this, and a number of actual and proposed uses were reported. These included the use of UAVs to collect patient samples and deliver to diagnostic centres and the distribution of vaccines [65] to difficult access communities [66]. The practicalities of UAV use for remote diagnosis and patient data collection have been reported [67] and also the screening of large populations with thermal imaging to detect pyrexia [68]. Once public health measures are in place UAVs can be used to monitor the effect of outside social distancing policies and social mixing [69, 70]. UAVs have even been proposed for effective spraying of antiviral agents to outdoor areas to prevent infection [71].

### Threats and hazards posed by UAVs

This article is focussed on the potential use of UAVs to enhance provision of care in the pre-hospital environment. Another potential interface between pre-hospital practitioners and the use of UAVs are those where UAVs are deliberately used to inflict harm. This may result in pre-hospital responders either treating victims of UAV attacks or being at risk from them. A review of the global terrorism database reported 76 terrorist attacks using UAVs between 2016 and 2019 [72]. Attacks were increasingly frequent and, although less lethal than standard explosive attacks, likely to be an increasing threat [73]. Some attacks have been very effective and received international attention because of the threat to life and infrastructure that this technology can pose. The attack and disruption of the Aramco oil installations in Saudi Arabia in 2019 [74] and the use of UAVs to disrupt shipping in the Red Sea [75] are a recent examples. The potential for non-conventional attacks is also present. UAVs could be adapted to deliver chemical or biological agents to large numbers of the population. Some other specific threats have been identified including the use of highly potent opioids to produce large numbers of casualties [76]. Another threat which has been identified is the interference, theft or malicious re-tasking of UAVs used by the authorities or EMS providers because of software security vulnerabilities [77].

A much more common issue in pre-hospital operations is the avoidance of unauthorised UAVs when operating air ambulances. Numerous incidents have been reported in the press and to regulatory aviation authorities. The risks are high because air ambulances frequently fly unplanned missions in urban areas at low altitude– the same environment where UAVs are most frequently encountered.

### Summary

This review has summarised how UAV commercial technology has translated into a variety of roles that can deliver and support pre-hospital clinical care. The move from proposal and simulation of applications to real world use is short and this is reflected in the real uses described in this review which had only been simulated or theoretical in similar reviews written only a few years ago. Publications related to the use of UAVs are expanding rapidly although most studies are descriptive or observational. High quality evidence is scarce. A well constructed recent systematic review of the available evidence to support the use of UAV’s in emergency health care documented only six studies which were RCTs or quasi experimental [78]. Three of the identified studies demonstrated reduced time to location of patients, two demonstrated reduced time to perform triage and two examined cost-effectiveness of sample transport in difficult terrain and showed benefit for longer distance transports. Rapid developments are driven by demand and this is illustrated in the considerable advances made in recent conflicts and the COVID– 19 pandemic.

UAV use is now established in pre-hospital emergency medicine roles and will likely significantly increase as UAV technology and battery endurance improves and regulatory challenges are addressed. Unmanned patient and rescuer transport in certain circumstances also now seems inevitable in the not too distant future.

### Limitations

The material about UAVs and their applications to pre-hospital medicine are distributed widely in technical and non-medical sources as well as the mainstream medical literature. The search strategy conducted may have missed some relevant material and the authors judgement of what is ‘relevant’ may be subjective. In addition we have included some material which we believe is very relevant to pre-hospital practice but may not directly influence the practice pre-hospital interventions. This may include the use of UAVs to assess scenes and locate patients by other EMS services.
